# Case report: Acute vestibular syndrome and cerebellitis in anti-Yo paraneoplastic syndrome

**DOI:** 10.3389/fneur.2022.960584

**Published:** 2022-08-26

**Authors:** Bassil Kherallah, Elias Samaha, Sarah E. Bach, Cindy Guede, Jorge C. Kattah

**Affiliations:** ^1^Department of Neurology, University of Illinois College of Medicine, Peoria, IL, United States; ^2^Department of Pathology, University of Illinois College of Medicine, Peoria, IL, United States

**Keywords:** acute vestibular syndrome (AVS), cerebellitis, PCA-1, anti-Yo, paraneoplastic syndrome

## Abstract

**Background:**

We define acute vestibular syndrome (AVS) as a sudden onset vertigo, nausea, vomiting, and head motion intolerance, more frequently associated with an acute peripheral and unilateral vestibulopathy. About 10–20% of all cases with central vestibulopathy are secondary to stroke. We report three patients evaluated over the past decade with an acute AVS along with subtle downbeat nystagmus (DBN), followed by dysarthria and progressive truncal and limb ataxia, as well as increasing DBN intensity.

**Methods:**

All patients underwent neurologic examination, video-oculography, MRI, serum cancer markers, spinal fluid examination, paraneoplastic panel testing, and oncologic workup. With a consolidated diagnosis of cancer/paraneoplastic syndrome, we treated with plasma exchange (PLEX), high-dose steroids, surgery, and oncologic investigation. We additionally provided oncotherapy in one out of three patients.

**Results:**

All three patients had an acute AVS, downbeat nystagmus DBN, and inability to perform tandem gait. Two of three patients had a normal head impulse test (HIT). As acute vertigo, nausea, and vomiting subsided, a progressive cerebellar syndrome ensued characterized by persistent DBN, impaired horizontal and vertical pursuit, impaired VOR suppression, truncal and limb ataxia, and dysarthria. All patients had normal MRI brain studies excluding stroke. CSF studies demonstrated lymphocytic pleocytosis and elevated protein. One patient had confirmed ovarian cancer with high CA-125 serum levels; another had undifferentiated cancer of unknown primary with high CA-125 and one patient with esophageal cancer. All had a positive PCA-1 antibody titer, also known as anti-Yo antibody. In one patient with expeditious immunosuppression, the ataxia progression slowed for 18 months, whereas the other two patients with delayed initiation of treatment had more rapidly progressive ataxia.

**Discussion:**

Paraneoplastic encephalitis related to PCA-1 antibody (Anti-Yo) targets Purkinje cells and cells in the granular layer of the cerebellar cortex. Clinically, our patients had a central AVS characterized by DBN and followed with progressive ataxia and unremarkable neuroimaging studies. Rapid initiation of treatment may offer a greater chance to prevent further neurologic decline. Any patient with an AVS as well as DBN and normal MRI should have an expeditious workup to rule out metabolic, toxic, and infectious causes just prior to considering prompt treatment with high-dose steroids and plasma exchange (PLEX) to mitigate the risk of rapidly progressive and irreversible neurologic decline.

## Introduction

Paraneoplastic cerebellar degeneration (PCD) is a rare neurologic condition most commonly due to anti-Purkinje cell cytoplasmic antibody type-1 (PCA-1), also commonly referred to as anti-Yo antibody, which is typically associated with breast and ovarian cancers (1). Neurologic manifestations often predate diagnosis of malignancy (2). Immune-mediated damage to Purkinje cells (PC) is typically irreversible, often resulting in permanent devastating disability in afflicted patients (3). Typical targets in PCA-1 antibody-mediated damage involve PC and granular cells in the cerebellar cortex, with secondary demyelination of the dentate nucleus (4, 5). Even though there is no specific mention of PC loss in the flocculus, midline uvula, and nodulus, it is predictable that these structures were involved in n =19/55 downbeat (DBN) anti-Yo patients (5). In this report, we highlight a common anti-PCA-1 phenotype with an acute vestibular syndrome (AVS), defined as the sudden onset of vertigo, nausea vomiting, and head movement intolerance (6, 7) due to a central lesion. The main features include subtle DBN while looking straight ahead and rapid progression from grade 1 to grade 3 truncal ataxia, with an inability to stand or sit without support. Nausea, vomiting, and vertigo rapidly improved in all three patients within two weeks. A normal MRI combined with an inflammatory CSF pointed to a diagnosis of acute cerebellitis. The differential diagnosis included an infectious, para-infectious, or paraneoplastic etiology (8). In the largest previous series of 55 anti-Yo antibody syndrome patients, 20 had acute continuous vertigo, only a few had nausea and vomiting, evolving within days to subacute ataxia, and the remaining patients had a picture of acute cerebellitis, and 19 of their 55 patients had DBN (5).

To date, current literature provides evidence for prompt plasma exchange treatment (PLEX) in halting the progression of cerebellar symptoms in some cases (9) with greater survival length and quality of life. Our reported cases and literature reviews suggest that a prompt combination of PLEX and high-dose steroid treatment in patients with characteristic clinical and CSF findings with normal MRI is justified. Here, the goal realistically at this time is rapid treatment initiation to prevent further neurologic decline, even in the absence of confirmed serologic antibodies.

## Methods

All patients had video recordings at the bedside performed by trained doctors of Audiology, advanced nurse practitioner, or registered nurse (RN). We initially recorded the VOG with a target placed at 1 m from the patient and used Otometrics (CHARTR 200 goggles). Recordings began with fixation straight ahead and eccentric right, left, up, and down gaze positions, followed by saccade and pursuit eye movements as the patient tracked the examiner's finger. We then lowered the shield of the goggles to study the possibility of suppressed spontaneous nystagmus. In patient 2, we switched the initial goggles to the lighter Otometrics (Natus Video-Head Impulse goggles) to test and record the horizontal (h) head impulse test of the right eye and to screen for skew deviation during alternate cover test, as the patient fixated at a central target (HINTS protocol). We recruited patient 1 in 2010 before we had the ability to quantitate the VOR. We evaluated the second patient in 2017. The third patient in 2022 declined video head impulse testing (vHIT). Patient 1 had a paraneoplastic panel performed by Athena Labs. Patients 2 and 3 had antibodies tested by Western blot, at the Mayo Clinic (Rochester, MN). The paraneoplastic panel included anti-GAD 65 antibodies. All patients had MRI brain neuroimaging and spinal fluid analysis, followed by investigation of an underlying neoplasm, and biopsy of neoplastic tissue after we classified the presenting AVS as the initial phase of a progressive ataxia syndrome.

## Patient 1

**Figure 1:**
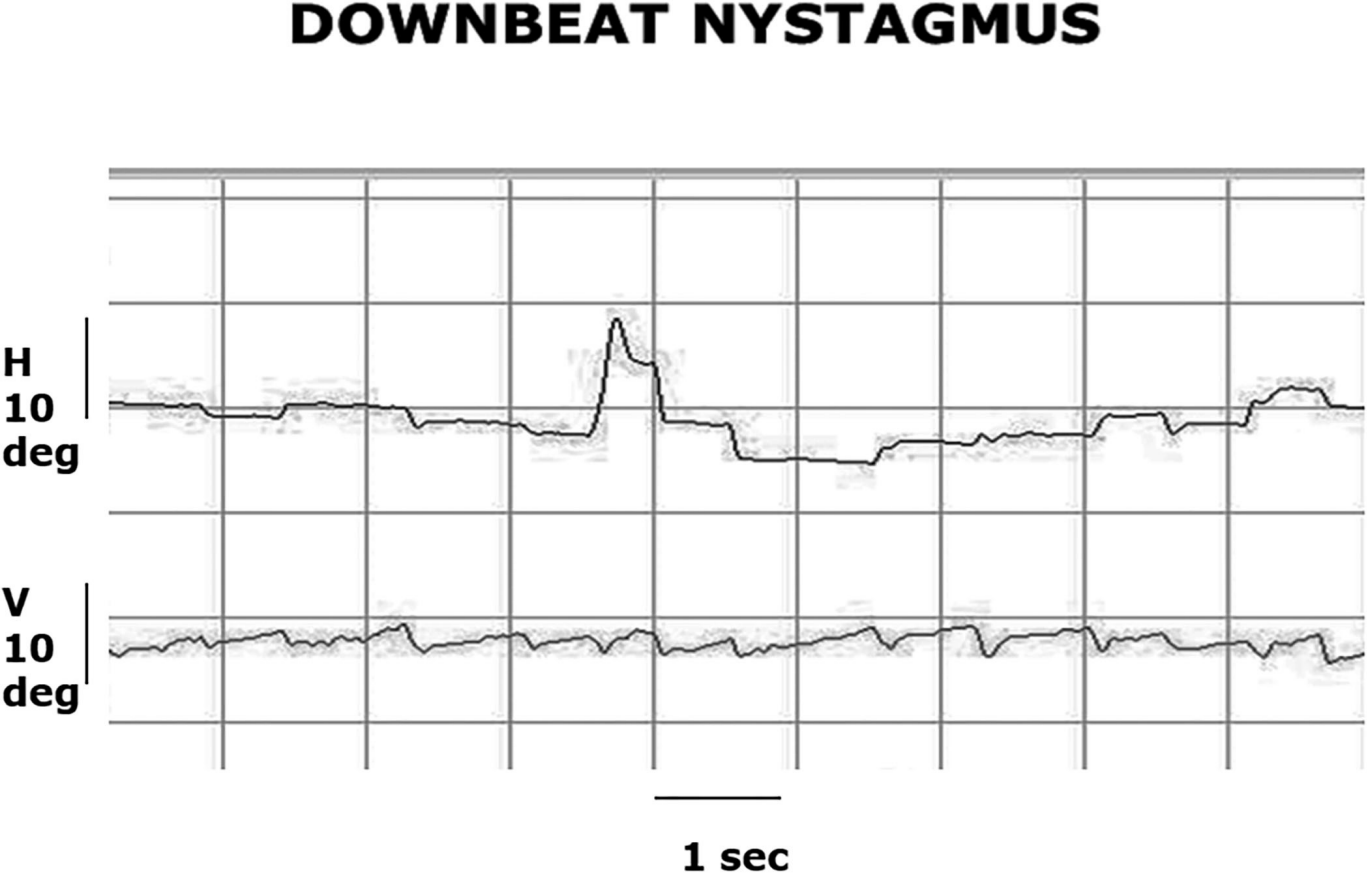
Video-oculography (VOG) recording of central fixation. The patient has a primary gaze, low-amplitude downbeat nystagmus with a slow phase velocity of 2 deg/sec and a frequency of 2 Hz, and this nystagmus increases in amplitude and velocity in right and left gaze.

A 67-year-old woman presented with acute continuous vertigo with associated nausea and vomiting. We admitted the patient for neurologic evaluation due to substantial truncal ataxia and inability to sit without support. Her posture, gait difficulty, evolved over 5 days from an initial wide base stance on admission. Two serial stroke protocol MRI brain studies with gadolinium within 48 h were performed, both with normal results. Otoneurology consult performed one week after symptom onset (Supplementary Video 1, patient 1) identified low-amplitude DBN best seen with ophthalmoscopy. Furthermore, she had direction-changing horizontal (h) nystagmus in right and left gaze with a downward and oblique direction; she had saccadic h and vertical (v) pursuit (Supplementary Video 1, Figure 1), mostly associated with DBN (Table 1); she had failed to suppress the VOR with visual fixation (VFX). Superimposed DBN on the pursuit tracings interfered with pursuit gain analysis. The head impulse test (HIT) was normal (Table 1, patient 1). We were unable to record the vHIT in 2010. Initially, there was no significant limb dysmetria or dysarthria. CSF studies demonstrated lymphocytic pleocytosis (98 /mm^3^) and elevated protein (106 mg/dL). The diagnostic differential suggested an infectious, para-infectious, or a paraneoplastic syndrome. Additional diagnostic studies included a paraneoplastic panel, CT chest/abdomen, CA-125, and obstetrics/gynecology consult. CT identified an ovarian mass, and CA-125 was significantly elevated at 3,703 μ/mL. Parallel to the diagnostic effort, she began 1 g IV methylprednisolone daily, followed by five sequential, daily plasma exchange (PLEX) treatments. Subsequently, she underwent complete surgical excision of the identified ovarian mass, which yielded a diagnosis of ovarian carcinoma. In 10 days, the paraneoplastic panel result was positive for PCA-1 antibodies.

**Table 1:** AVS characteristics preceding ataxia in anti-yo antibody syndrome.

**Age/gender**	**Duration of acute vertigo, nausea and vomiting**	**Straight ahead nystagmus SPV**	**Lateral gaze nystagmus**	**Head impulse test**	**Other^∧^**
Female 67	15 days	DBN ^*^ 2 deg/sec	R: h/RBN + DBN L: h/LBN + DBN	Normal^**^	Wide base stance. Could not do tandem gait by history
Male 68	1 week	DBN 3 deg/sec	R: h RBN/DBN L: h LBN/DBN	RL: 0.57 ± 0.05 LL: 0.89 ± 0.05 LA: 0.95 ± 0.11 RP: 0.57 ± 0.08 LP: 0.69 ± 0.13 RA: 1.44 ± 0.13	Could not sit up without support Skew. R hypotropia
Female 80	1 week	DBN 3 deg/sec	R: h RBN/DBN L: h LBN/DBN	Normal^**^	Wide base stance. Could not do tandem gait

DBN, Downbeat nystagmus; h-RBN, Right beat nystagmus; h-LBN, Left beat nystagmus; SPV, slow phase velocity.

* Subtle nystagmus on straight gaze visible with ophthalmoscopy or magnification from Frenzel or Video goggles.

** Clinical head impulse.

∧ Wide base stance rapidly evolved to inability to sit in patients 1 and 2, patient 3 had a slower transition according to records, when first examined she could not sit without support.

After surgery, treatment with carboplatin partially resulted in improvement and resolution of her AVS within 2 weeks despite persistent DBN; however, visual acuity was not significantly impaired. Gait remained unstable, unable to ambulate beyond a few steps independently, and continued to depend on a wheelchair for mobility. Follow-up CA-125 decreased to 111 U/mL after initial treatment. She remained neurologically stable for about 2 years. Unfortunately, due to later tumor recurrence with systemic metastases including supratentorial brain lesions, she elected to withdraw life-prolonging treatments and transitioned to hospice cares. At her last visit with neurology, she did not have neurologic deficits outside her known cerebellar abnormalities.

**Figure 2:**
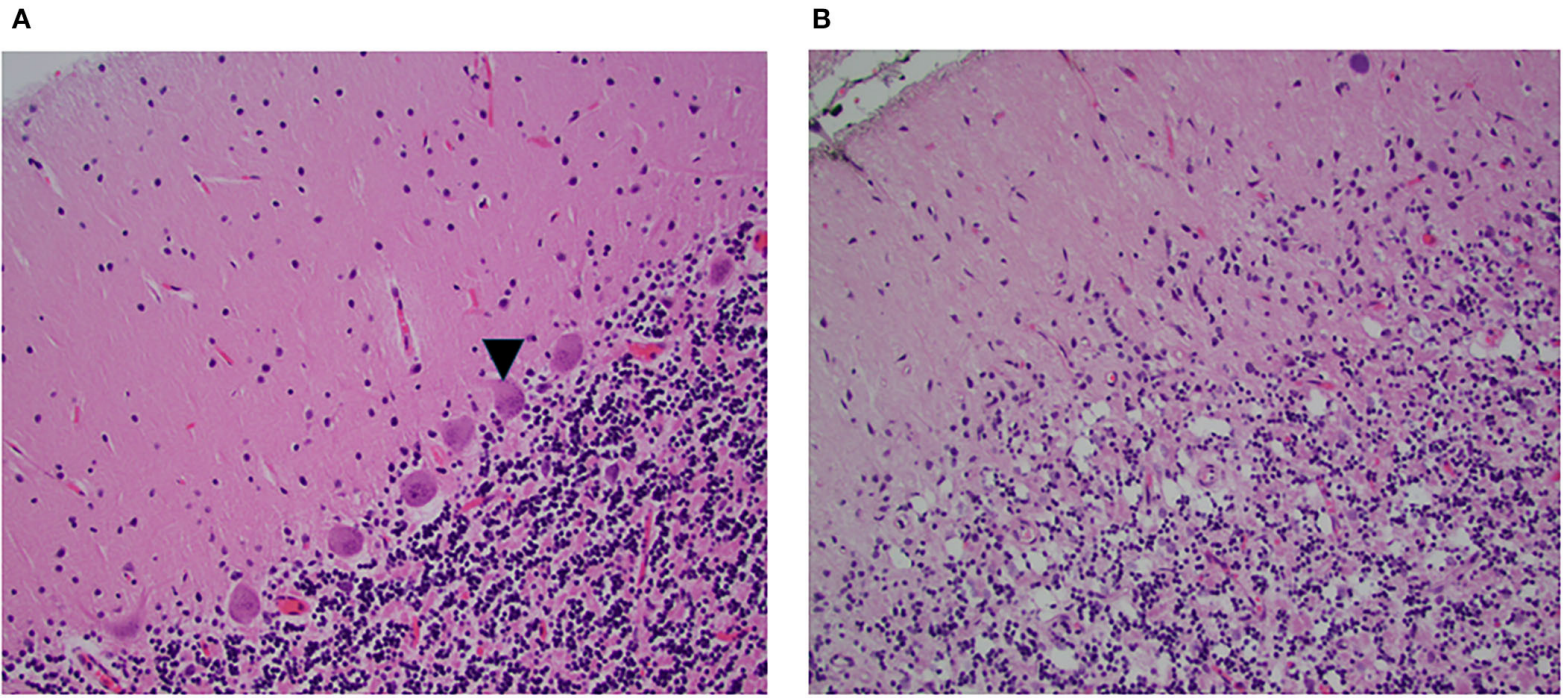
Microscopic examination of the hemispheric cerebellar cortex. H&E x20. **(A)** Normal cerebellar cortex, the arrow points to morphologically intact Purkinje cells. **(B)** Similar section inpatient 1 shows absent Purkinje cells, note absent of acute inflammation. In this patient, the deep nuclei were normal.

The autopsy was significant for extensive cerebellar folia atrophy and loss of PCs in the midline and lateral cerebellar hemispheres (Figure 2). In addition, she had cerebral hemisphere metastases of ovarian origin. Of note, there was no evidence of active inflammation.

## Patient 2

**Figure 3:**
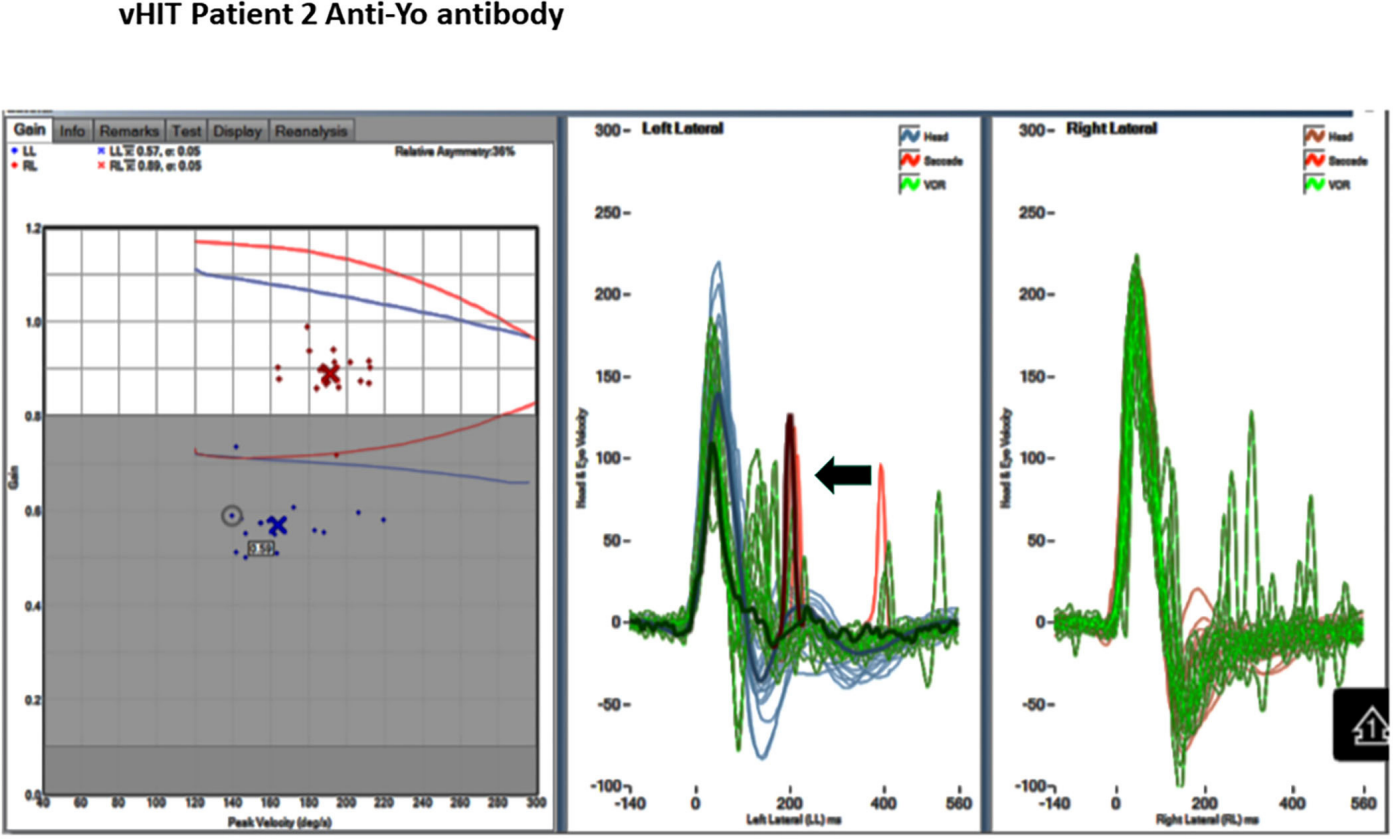
Video head impulse test (vHIT) of the horizontal VOR. Gain: 0.57 right and 0.89 left. Note catch-up saccade. There is a catch-up saccade 100 m sec after the end of the left head impulse (arrow).

A 68-year-old man presented as a transfer from an outside hospital with acute continuous vertigo, nausea, vomiting, oscillopsia, and ataxia with worsening ability to stand over the week prior to admission. Examination on arrival was notable for subtle DBN when looking straight ahead, more prominent in lateral gaze, and mixed with a direction-changing horizontal component in lateral gaze (Table 1, patient 2). We found DBN superimposed on the horizontal and downward pursuit and poor VFX of rotational nystagmus, and he had decreased VOR gain and overt corrective saccades in response to a left head impulse test (Figure 3). He could not sit without support and had bilateral upper and lower extremity ataxia. The nausea and vomiting subsided with medication; however, DBN increased in intensity. There were no signs of elevated intracranial pressure or meningeal irritation. A head MRI was normal making the likelihood of PRION unlikely.

The patient underwent evaluation for infectious, para-infectious, and paraneoplastic etiologies. CSF studies revealed lymphocytic pleocytosis (50 cells/1 mm^3^, 100% lymphocytes), and protein mildly elevated 54.5 mg/dL (Table 1, patient 2). Treatment with high-dose steroids and PLEX began. Oncologic workup was significant for a distal esophageal mass, and biopsy showed poorly differentiated adenocarcinoma of the distal esophagus. A paraneoplastic panel was positive anti-PCA-1 antibodies. In the rehabilitation unit 2 weeks later, the patient continued to deteriorate rapidly with progressive confusion, diaphragm, and generalized truncal tremor/myorhythmia syndrome. He elected no further treatment and later transitioned hospice care.

## Patient 3

An 80-year-old previously healthy Caucasian female presented as a transfer from an outside hospital for a one-month history of new rapidly progressive dysarthria and ataxia. Of note, we did not see her during the initial phase of her illness. Past medical history was significant for ulcerative colitis and hysterectomy with unilateral oophorectomy for reported symptomatic fibroids during her adolescent years. Initial symptoms were sudden onset unprovoked continuous vertigo, generalized weakness, and nausea with vomiting. According to her description and chart records within days of symptom onset, while her nausea, vomiting, and vertigo improved, her speech function rapidly deteriorated with slow and slurred speech, along with continued deterioration of her ability to ambulate due to ataxia.

Notable exam findings included delayed vertical saccades and subtle fixation DBN, more prominent on left and down gazes, mixed with a horizontal direction-changing nystagmus, as well as dysarthria best described as a monotonous scanning speech, with slow articulation and variable pitch and loudness. Generalized and non-lateralizing weakness developed with age-appropriate deep tendon reflexes and sensory examination. In addition, the patient had moderate ataxia of all extremities but more prominent on her left side.

Serial MRI brain with gadolinium studies performed on initial symptom onset, 1-week post-onset, and 2-week post-onset was unremarkable with no interval findings to correlate with symptom progression. CSF analysis 1-week post-symptom onset was significant for elevated protein (118 mg/dL) and lymphocytic pleocytosis (31 WBC/mm^3^, 95% lymphocytes, and 4/mm^3^ RBC). CSF glucose was 43 mg/dL. Incidental finding of St. Louis Encephalitis IgG with titer 1:4 (negative IgM), however, PCA Ab type-1 was positive with significant titer of 1:6,1440 (normal <1:240).

Preceding her transfer from the referring hospital, attempts to treat for suspected paraneoplastic rhombencephalitis with a 5-day course of high-dose solumedrol alternating with IV immunoglobulin treatment days, with inconsistent neurologic improvement and an overall continued steady decline of independent mobility, speech, and function.

Oncologic workup done prior to transfer included transvaginal pelvic ultrasound as well as CT chest, abdomen, and pelvis with contrast all of which were unremarkable for suspicious masses or fluid collection. Serum CA-125 marker was elevated at 183.6 U/mL, with borderline CEA elevation at 3.1 ng/mL.

During her hospitalization, we treated her with a 5-day course of PLEX every other day alternating with a second course of high-dose methylprednisolone. However, there was minimal recovery and functional improvement on this treatment, the patient stabilized with no further functional decline and progression in symptoms after initiating plasmapheresis. PET scan was performed while inpatient and demonstrated multiple hypermetabolic, non-enlarged peri-esophageal, gastro-hepatic, and periaortic lymph nodes. Initial lymph node biopsy was notable for non-small cell carcinoma of unknown primary. CA-125 repeat level was 234 U/mL. Fine-needle aspiration of lymph node showed non-specific malignant neoplasm pathology (possibly pancreatic–biliary or upper GI tract origin), though colonoscopy and MRI abdomen were all non-diagnostic. After completing plasmapheresis treatment, the patient started on initial maintenance rituximab infusion; however, we discontinued life-prolonging treatments following patient and family's directive to transition to hospice due to severity of her persistent disability.

## Discussion

The most common cause of an AVS is an acute vestibular neuritis (or acute vestibulopathy) (10), whereas the most common central cause is stroke. We hope that introducing awareness of additional central causes, albeit uncommon, increases the diagnostic accuracy of clinician evaluating acute vertiginous patients. Here, the otoneurologic examination was sensitive to central localization and provided compelling evidence to proceed investigating a specific non-vascular etiology. For organization purposes, we will discuss the initial AVS characteristics in these patients, followed by comments on their subsequent progressive ataxia syndrome.

### AVS: An initial manifestation of anti-Yo antibody syndrome

Continuous vertigo associated with nausea, vomiting, and head movement intolerance lasting for a prolonged period (longer than 72 h) is most frequently associated with an acute vestibular neuritis (acute unilateral vestibulopathy). The cause for an AVS is peripheral in most patients, and about 5–15% of cases have unilateral central lesions. The symptoms cannot provide a peripheral vs. central localization diagnosis, and the physical examination identifying “central signs” provides the localization/lateralization information (6, 7). In two of our patients, subtle DBN in straight gaze was initially detected using ophthalmoscopy and goggles, increasing in lateral gaze and mixed with a horizontal direction-changing nystagmus, suggesting central localization in either the cerebellum or brainstem (7). None of our patients had a metabolic abnormalities, infectious cerebellitis, or medication effects to cause DBN; thus, the nystagmus direction here was the only initial overt and unequivocal central localization abnormality, particularly with normal brain MRI. In addition, patients 1 and 3 had a normal HIT, indicating preserved horizontal VOR gain (Table 1). Patient 2 had unilaterally decreased VOR gain (Table 1, Figure 3), while this combination points to combined peripheral/central etiology. In this context, it is probably secondary to vestibular nuclear or a floccular abnormality (11, 12).

Unlike peripheral cases of AVS, as nausea/vomiting and vertigo subsided, our patients worsened with increasing DBN and truncal ataxia as well as new signs of developing generalized cerebellar dysfunction.

All patients underwent a second MRI to exclude a stroke before formal audiology evaluation was done. Because of the subacute progressive ataxia, despite resolution of the AVS symptoms, we considered the possibility of rhombencephalitis, either viral, autoimmune, or paraneoplastic. Our patients did not have initial upbeat nystagmus (UBN) switching to DBN, as seen frequently in Wernicke's encephalopathy (13) or magnesium depletion (14). The obvious next step was an examination of the CSF and serum paraneoplastic/autoimmune investigation. Normal MRI of the brain in sequence excluded demyelinating diseases such as neuromyelitis optica spectrum disorders (15) and infectious etiologies. Finally, prion disorders with some frequency begin with rapidly progressive ataxia (7) and rarely with an AVS as well (16).

### Subacute progressive ataxia syndrome

Once the AVS resolved, unexpectedly, the truncal ataxia increased, the nystagmus did not resolve, and additional signs of cerebellar dysfunction such as dysarthria and limb ataxia developed, by this time the CSF showed a lymphocytic pleocytosis, without evidence of an infectious process. The CT scan of chest/abdomen revealed abnormal mass lesions in all three patients, with biopsy confirmation of cancer and a positive paraneoplastic panel with anti-PCA-1 (Anti-Yo) antibodies detected by Western blot. Our patients fit the diagnostic criteria for paraneoplastic neurologic syndromes as described in 2004 (17).

The clinical manifestations of the anti-Yo antibody syndrome are consistent particularly because of the dysimmune response targeting specific neurons in the cerebellum, thus manifesting a central AVS associated with DBN, followed by an acute or a subacute progressive cerebellitis, typically in women with normal neuroimaging, inflammatory CSF findings, and increased CA-125 levels. In retrospect, initial dysfunction of the flocculus explains the DBN, in addition to the worsening gaze holding failure, abnormal pursuit, and impaired VFX (12) noted in our three patients. On occasion, the HIT may be abnormal as well, as noted in patient 2, rarely a finding in lesions of the flocculus (11). Anti-Yo antibody syndrome is extremely uncommon in men (18), most associated with cancer of the ovary or other gynecologic malignancies (5). The differential diagnosis includes infectious or post-infectious cerebellitis, other paraneoplastic syndromes, and prion diseases (5). Obviously, lymphocytic pleocytosis excludes the latter.

Nausea and vomiting are common in AVS, most attributed to asymmetric firing of vestibular nuclei neurons in the medial vestibular nucleus leading to motion sickness due to activation of the nucleus solitarius, the reticular formation, and the parabrachial nucleus (19). In patient 2, this was the likely mechanism, as the h-VOR gain was asymmetric. In anti-Yo, an alternative mechanism of nausea and vomiting could be secondary to involvement of the area postrema, which is specifically demonstrated in neuromyelitis optica (20). Asymmetric disinhibition of vestibular nuclei is a possibility (6, 7). In general, nausea and vomiting in our patients lasted longer than the average duration associated with vestibular neuritis (6). In the Peterson series, vomiting was infrequent (5).

Anti-Yo antibody syndrome is infrequent in men and raises concern for a false-positive result (17, 18). Our patients #2 and #3 were tested with Western blot and reported by the Immunology Laboratory of the Mayo Clinic, Rochester, MN, and correlated with a clinically consistent AVS. Importantly, there are a handful of preceding reports identifying this antibody in men, at least two patients with GI adenocarcinoma, one in the stomach with post-mortem identification of the anti-Yo antibody in the neoplasm, and a second report the same year in association with a neoplasm of the esophagus (3, 21). Moreover, our patients had a neurologic syndrome preceding the diagnosis of cancer, initially associated with an AVS, followed by ataxia. At minimum, this presentation is clinically consistent with the current diagnostic criteria for a paraneoplastic syndrome (17) and is consistent with serologic anti-Yo antibody positivity in the absence of alternate causes excluded by comprehensive evaluation. In addition, the rhythmic movement of the abdominal wall is potentially related to inferior olivary nucleus deafferentation from dentate nucleus involvement. Confusion develops frequently in anti-Yo syndrome patients (5).

The neuropathology of this syndrome relates to loss of PCs and other cerebellar neurons in the granular layers of the cerebellum (5). In patient 1, the autopsy showed loss of PCs, with demyelination of the dentate nuclei (Figure 2). The anti-PCA-1 antibody binds to a CDR2 protein involved in transcription (3). Eventually, there is a severe and disabling ataxia with an inability to sit without support. The particularly aggressive course in patient 2 may possibly relate to the uncommon gender and cancer association (18). The fact that the antibodies bind to nuclear rather than cell surface or synaptic antigens (22) may possibly explain a decreased therapeutic response to available treatment. Rarely, rapid tumor extraction, oncotherapy, and immunosuppression lead to improvement of symptoms (5).

One of our cases, who consistently demonstrated a therapeutic response, suggests a more favorable neurologic prognosis with earlier treatment following onset of AVS, the reported mechanism of PCA-1 causing irreversible injury to PCs theoretically slows down with timely intervention. The profound degeneration of the cerebellum associated with PCA-1, noted in the pathologic examination in patient 1, explains the poor neurologic prognosis. Thus, our series highlights the importance of prompt treatment in aborting the disease process and attempting to treat the underlying cancer. Definitive oncotherapy remains insufficient. Tumor recurrence led patient 1 to discontinue treatment; at autopsy, she had severe cerebellar degeneration and diffuse ovarian cancer metastases.

Both our experience and the literature in PCA-1 aim to improve awareness of paraneoplastic disorders. As current data shows greater and longer quality of survival, relating to early treatment, in an otherwise devastating condition, with has unchanged prognosis for the last three decades (5). Recent development in the treatment of cancer with checkpoint inhibitors has not shown effectiveness yet in epithelial ovarian carcinoma (23). It is possible that in the near future, an expeditious diagnosis will be critical. Checkpoint inhibitors theoretically may worsen neurologic abnormalities, which may warrant development of specific protocols to preserve the anti-neoplastic response while modulating the co-existent neurologic syndrome.

As previously mentioned, our best survival outcome possibly relates to expeditious treatment. In patient 1, slow neurologic deterioration for at least 18 months post-symptom onset enabled her to regain independent function abilities. Currently available maintenance options include rituximab, cyclophosphamide, and corticosteroid treatment; formal guideline is not yet established. Obviously, these measures were implemented in concert with surgical tumor resection and proper oncotherapy.

The main limitation in this paper is the small number of patients. In addition, the fact that patient 2 is a male with a paraneoplastic syndrome raises the possibility of an alternative antibody. However, the association between anti-Yo paraneoplastic syndrome and esophageal cancer was previously reported (21). Finally, we examined patient 3 with formal audiology evaluation after the illness initial phase had resolved. This limitation was countered by the patient providing reliable history along with detailed extensive neurologic documentation on record and collateral history provided by family.

The second most common paraneoplastic antibody associated with ataxia is probably anti- ANNA-1 (Anti-Hu) (24), and the anti Kelch-11 protein antibody is associated with small cell cancer of the lung and testicular seminoma, germinomas, or teratoma (25). The latter is associated frequently with sensorineural hearing loss and episodic vertigo, DBN, and progressive ataxia.

In conclusion, the PCA-1 (anti-Yo) immunophenotype is very characteristic, and the pathogenesis of the syndrome remains unclear; therefore, a promising disease-modifying treatment is not yet available, except at best to cure the cancer and stop neurologic deterioration. Future approaches should consider the clinical characteristics.

## Data availability statement

The original contributions presented in the study are included in the article/Supplementary material, further inquiries can be directed to the corresponding author.

## Ethics statement

Written informed consent was obtained from the individual for the publication of any potentially identifiable images or data included in this article.

## Author contributions

ES: direct patient evaluation, hospital care and article review, and edits. SB: processed autopsy samples to create original pathology slides. CG: video frenzel goggle testing and nystagmography. JK: direct manuscript editor, performed examination in all three subjects, and analyzed their eye movement recordings. All authors contributed to the article and approved the submitted version.

## Conflict of interest

The authors declare that the research was conducted in the absence of any commercial or financial relationships that could be construed as a potential conflict of interest.

## Publisher's note

All claims expressed in this article are solely those of the authors and do not necessarily represent those of their affiliated organizations, or those of the publisher, the editors and the reviewers. Any product that may be evaluated in this article, or claim that may be made by its manufacturer, is not guaranteed or endorsed by the publisher.

## Supplementary material

Supplementary Video 1 Recording of eye movements with the use of Frenzel glasses to block fixation. The patient has low-amplitude, slow velocity DBN that increases in lateral gaze in amplitude and velocity.
